# An Insight into the Sex Differences in COVID-19 Patients: What are the Possible Causes?

**DOI:** 10.1017/S1049023X20000837

**Published:** 2020-06-18

**Authors:** Parisa Maleki Dana, Fatemeh Sadoughi, Jamal Hallajzadeh, Zatollah Asemi, Mohammad Ali Mansournia, Bahman Yousefi, Mansooreh Momen-Heravi

**Affiliations:** 1. Research Center for Biochemistry and Nutrition in Metabolic Diseases, Institute for Basic Sciences, Kashan University of Medical Sciences, Kashan, Islamic Republic of Iran; 2. Department of Biochemistry and Nutrition, Research Center for Evidence-Based Health Management, Maragheh University of Medical Sciences, Maragheh, Iran; 3. Department of Epidemiology and Biostatistics, School of Public Health, Tehran University of Medical Sciences, Tehran, Iran; 4. Stem Cell Research Center, Tabriz University of Medical Sciences, Tabriz, Iran; 5. Department of Biochemistry, Faculty of Medicine, Tabriz University of Medical Sciences, Tabriz, Iran; 6. Department of Infectious Disease, school of medicine, Kashan University of Medical Sciences, Kashan, Islamic Republic of Iran; 7. Infections Diseases Research Center, Kashan University of Medical Sciences, Kashan, Islamic Republic of Iran

**Keywords:** ACE2, COVID-19, estrogen, gender, sex differences, sex hormones, testosterone

## Abstract

Studies have reported a sex bias in case fatalities of COVID-19 patients. Moreover, it is observed that men have a higher risk of developing a severe form of the disease compared to women, highlighting the importance of disaggregated data of male and female COVID-19 patients. On the other hand, other factors (eg, hormonal levels and immune functions) also need to be addressed due to the effects of sex differences on the outcomes of COVID-19 patients. An insight into the underlying causes of sex differences in COVID-19 patients may provide an opportunity for better care of the patients or prevention of the disease. The current study reviews the reports concerning with the sex differences in COVID-19 patients. It is explained how sex can affect angiotensin converting enzyme-2 (ACE2), that is a key component for the pathogenesis of COVID-19, and summarized the gender differences in immune responses and how sex hormones are involved in immune processes. Furthermore, the available data about the impact of sex hormones on the immune functions of COVID-19 cases are looked into.

## Sex Differences and COVID-19

Several studies have shown that there is an association between the sex of COVID-19 patients and fatality rates, as well as critically-ill status. Studies in China, South Korea, United States, and Italy have reported a similar trend of sex bias in fatality rates of COVID-19 patients, representing that case fatality rates are higher in male patients than in female patients.^1-3^ Noteworthy, the same findings have reported in the previous outbreaks of coronaviruses, Severe Acute Respiratory Syndrome (SARS), and the Middle East Respiratory Syndrome (MERS).^4,5^ Moreover, studies performed on mice have shown that males are more susceptible to coronaviruses.^6^ The likelihood of death is reported to be 65% higher in male patients with COVID-19 than in women. The World Health Organization (WHO; Geneva, Switzerland) indicated that a lower percentage of women who are infected with the virus will die in comparison with men (1.7%/2.8%). Another investigation also reported that less female patients needed intensive care or died compared to male patients (15%/32%).^7^ In 43 hospitalized patients, women were significantly less prone to develop the severe form of the disease.^8^ Jin, et al^8^ expressed that male patients’ deaths in a population of 37 cases were 2.4-times that of female patients. Moreover, they concluded that men’s worse outcomes and higher deaths compared to women is not dependent on age.^8^ In a retrospective study of 168 confirmed patients with COVID-19, deaths occurred in 12.8% of men (n = 11/86); meanwhile, 7.3% of women died (n = 6/82).^9^ It is observed that there are differences in laboratory parameters of men and women, including neutrophil/lymphocyte ratio, hematocrit, hemoglobin, ferritin, alanine aminotransferase, aspartate aminotransferase, total bilirubin, C-reactive protein (CRP), blood urea nitrogen, and creatinine (all P values <.05). Moreover, male patients with comorbidities had a higher risk of developing a critically-ill status compared with men without comorbidities; whereas, there was no such association in women.^9^ Evidence suggested that female patients experienced some COVID-19 symptoms significantly more than male patients, such as fatigue, anosmia, headache, sore throat, and nasal obstruction.^10^ Moreover, some symptoms were experienced significantly more by men than women, including cough and fever. However, it is reported that there was no difference regarding disease duration between men and women.^10^ Borghesi, et al^3^ also reported that in COVID-19 patients aged between 50 to 79 years old, men had a significantly higher score of chest x-ray scoring system than women.

Besides the different effects of COVID-19 on the physical status of men and women, studies have shown that the same goes for mental health of people. Studies of general population indicated that women present more mental disorders symptoms compared to men during the COVID-19 pandemic, including signs of depression, anxiety, post-traumatic stress symptoms, and decreased sleep quality.^11,12^ In the viewpoint of health care workers, evidence shows that COVID-19 has affected the mental health of female medical staff more than men.^13^ Altogether, this information highlights the importance of disaggregated data of male and female COVID-19 patients. An insight into the underlying causes of sex differences in COVID-19 patients may provide an opportunity for better care of the patients or prevention of the disease. Additionally, attention should be paid to other factors which are or may be related to this sex bias in fatality rates of COVID-19 patients, including hormonal levels, immune functions, comorbidities, and related conditions, as well as gender-related factors, including lifestyle and socioeconomic aspects.^14^

## Possible Underlying Causes of Sex Differences in COVID-19 Patients

### Sex Differences in Inflammatory Processes

Since X-chromosome encodes some genes related to immune responses, women have a lower level of viral load and less inflammation compared to men. Women’s immune cells activate more than men, which is associated with the stimulation of toll-like receptor-7 and interferon production. Besides, after infection with viral agents, women produce lower levels of interleukin-6 (IL-6) compared to men, which is associated with better longevity.^15^ Plasma concentration of testosterone, which decreases with age, is reported to be reduced by some comorbidities, such as diabetes, obesity, and obstructive sleep apnea.^16^ Evidence has shown that these comorbidities are common in patients with COVID-19. Investigations have indicated that aging men have a reduced testosterone level, which is related to an elevated pro-inflammatory condition.^17^ Besides, it is observed that testosterone therapy leads to a reduction in IL-6, IL-1β, and tumor necrosis factor α (TNF-α).^18^ Therefore, Pozzilli, et al^19^ proposed that through the activation of cytokine storm, testosterone may be involved in the progression of COVID-19. Androgen receptor (AR) is involved in the regulation of both innate and adaptive immune responses, including the macrophage and neutrophil recruitment which is related to COVID-19.^20-22^ In mice infected with a respiratory virus, estrogen receptor (ESR) signaling is reported to increase the morbidity and mortality. While this observation applies to both sexes, estrogen therapy has resulted in the suppression of inflammatory processes and reduction of virus titers in animal models.^23^ Estrogen enhances immune responses and subsequently, pathogens clearance occurs faster and vaccines will be more effective. Contrary to estrogen, testosterone plays inhibitory roles in immune processes, which is a possible explanation for men’s higher susceptibility to infections.^24^ Thus, sex hormones’ alterations may cause some changes in the immune response to pathogen, indicating the role of some other relevant conditions, such as pregnancy and hormone therapies in COVID-19.^14^

In a cohort study of 31 male COVID-19 patients, data demonstrated that patients who were transferred to intensive care unit (ICU) or died in respiratory ICU (RICU) had lower amounts of total testosterone (TT) and calculated free testosterone (cFT) compared to patients who were transferred to internal medicine unit or were at a stable condition in RICU.^25^ The TT and cFT levels were negatively associated with some risk factors, including lactic dehydrogenase, procalcitonin, and neutrophil count. However, the levels of TT and cFT were positively associated with the lymphocyte count. Furthermore, TT had a negative correlation with the amounts of CRP and ferritin.^25^ Fagone, et al^26^ observed that in age group of 40 to 60 years, the transcriptomic characteristics of female lung tissue has more similarities to COVID-19-induced characteristics compared to male tissue. A possible explanation of the lower incidence of COVID-19 in female patients could be the lower threshold of acute immune response to COVID-19 in men compared to women. The AR regulates two chemotactic factors, the chemokine (C-X-C motif) ligand-1 (CXCL1) and chemokine (C-C motif) ligand-20 (CCL20), which are encoded by COVID-19-induced genes. The ESR1 regulates C3 and endothelin-1, which are also encoded by COVID-19-induced genes.^26^ Fagone, et al^26^ suggested that while CXCL1 and CCL20 are involved in different infections of coronavirus, response to these infections are occurred by different agent in men and women. Moreover, it is shown higher mortality rates in female mice with SARS coronavirus (SARS-CoV) who were treated by the antagonist of ESR in comparison with their counterparts who were treated with vehicle.^27^

### Angiotensin-Converting Enzyme-2 (ACE2) Differences in Men and Women

Angiotensin-converting enzyme-2 (ACE2) is an essential component for the entrance of COVID-19 to the cells.^28,29^ Since ACE2 gene is located on X-chromosome, women have the potential to be heterozygous regarding this enzyme while men are homozygous.^7^ To priming spike protein, COVID-19 also requires the transmembrane serine protease-2 (TMPRSS2).^30,31^ The TMPRSS2 is the only critical protease for the pathogenesis and viral spread of SARS-CoV.^32^ While TMPRSS2 gene transcription depends on the activity of AR, there is no other promotor to do the same function in humans.^33^ It is suggested that testosterone may lead to male predominance of severe infections (eg, COVID-19) through modulating the expression of TMPRSS2.^34^ In the other hand, TMPRSS2 is the most common gene involved in primary prostate cancer, indicating that administering inhibitors of TMPRSS2, which are currently used for prostate cancer, may be helpful for treatment or prevention of COVID-19.^33,35,36^ The ACE2 may be cleaved by TMPRSS2, leading to an enhancement in the entry of the virus.^37^ Interestingly, rebalancing of ACE1/ACE2 or high levels of ACE2 possibly enhances the COVID-19 outcomes in both males and females through reducing thrombosis, inflammation, and death.^7^

ADAM metallopeptidase domain-17 (ADAM-17), which is a disintegrin and metalloproteinase-17, leads to the soluble ACE2 (sACE2) and shedding of SARS-CoV through the cleavage of myocardial ACE2 (mACE2).^38,39^ Evidence has revealed that both sexes have the same levels of sACE2 up to the age of 12. However, its levels increase in boys with their growth and women have a lower level of sACE2 than men from the of 15.^40^ As evidenced by Sward, et al,^40^ cases with higher sACE2 (men>women) may have a greater risk of severe COVID-19. It is shown that ACE2 over-expression has led to the severity of SARS-CoV disease in mice.^41^ Studies also indicated that high activities of mACE2 and/or ADAM-17 may be involved in the severity of COVID-19.^38,41^ Contrary to the globally observed data, female rats with higher expression of ACE2 are reported to have poorer prognosis.^7^ Stelzig, et al^6^ reported that normal human bronchial epithelial (NHBE) cells that were treated with 17β-estradiol (E2) had a lower expression level of ACE2 messenger ribonucleic acid (mRNA) compared with NHBE cells that were treated with the vehicle, ethanol. Experimental animal studies have indicated that in kidney and adipose tissue, E2 enhances the activity and expression of ACE2.^42^ Noteworthy, precautions should be observed in testosterone therapy, especially in men with hypogonadism, due to the recent evidence of venous thromboembolism in COVID-19 patients.^42^

## Limitations

However, this study has some limitations. First of all, not all published papers are in English, and thus had to be excluded from this review. Another limitation that was faced was the inconsistency of existing data from COVID-19 patients. Besides, a significant number of studies concerning COVID-19 have not reported the disaggregated data of men and women due to the urgent need for rapid publications of the research. Furthermore, findings in this area are limited and there are not many large-scale studies reporting the sex differences in COVID-19 patients and the reason for this difference. Thus, further studies are required to draw a conclusion.

## Conclusions

Studies of COVID-19 patients have shown that men have a higher risk of developing to the severe form of the disease compared to women. Moreover, case fatalities are higher in male patients than female patients. Therefore, more attention should be paid to sex as an important variable. By reviewing the literature about the sex differences in COVID-19, it can be concluded that some sex-related factors are involved in determining the patients’ outcomes. This study shows that different levels of ACE2 in men and women, the effects of testosterone on ACE2 levels, and the fact that the ACE2 gene is located on the X-chromosome should not be ignored. Furthermore, the effects of sex on immune responses and data available on the sex differences in COVID-19 patients and immune system are summarized. However, findings in this area are limited and further studies are required to draw a conclusion.
